# Exocrine-to-endocrine differentiation is detectable only prior to birth in the uninjured mouse pancreas

**DOI:** 10.1186/1471-213X-10-38

**Published:** 2010-04-08

**Authors:** Daniel Kopinke, L Charles Murtaugh

**Affiliations:** 1 University of Utah, Department of Human Genetics, Salt Lake City, UT 84112, USA

## Abstract

**Background:**

Histological evidence suggests that insulin-producing beta (β)-cells arise in utero from duct-like structures of the fetal exocrine pancreas, and genetic lineage tracing studies indicate that they are maintained in the adult by self-renewal. These studies have not addressed the origin of the new β-cells that arise in large numbers shortly after birth, and contradictory lineage tracing results have been published regarding the differentiation potential of duct cells in this period. We established an independent approach to address this question directly.

**Results:**

We generated mice in which duct and acinar cells, comprising the exocrine pancreas, can be genetically marked by virtue of their expressing the mucin gene *Muc1*. Using these mice, we performed time-specific lineage tracing to determine if these cells undergo endocrine transdifferentiation in vivo. We find that *Muc1*^+ ^cells do give rise to β-cells and other islet cells in utero, providing formal proof that mature islets arise from embryonic duct structures. From birth onwards, *Muc1 *lineage-labeled cells are confined to the exocrine compartment, with no detectable contribution to islet cells.

**Conclusions:**

These results argue against a significant contribution by exocrine transdifferentiation to the normal postnatal expansion and maintenance of β-cell mass. Exocrine transdifferentiation has been proposed to occur during injury and regeneration, and our experimental model is suited to test this hypothesis in vivo.

## Background

The origin of pancreatic islet cells has been the subject of study and controversy since before the discovery of insulin [1-3]. Histological and ultrastructural studies suggested that islets arise from exocrine ducts during embryogenesis, but whether such exocrine-endocrine conversion continued after birth remained a matter of controversy [4]. Nucleotide analogues have recently been used to identify and trace the fate of proliferating cells, but these studies have been interpreted both for and against the hypothesis of new islet cell differentiation, or neogenesis, in adulthood [5,6]. Similarly, cell culture studies have provided evidence for and against neogenesis, but extrapolating these findings in vivo remains a challenge [2]. Nonetheless, determining whether β-cell neogenesis occurs in vivo will inform efforts to replenish β-cells lost in diabetes: if neogenesis can occur in mice, it might be possible in the human organ as well. Evidence against neogenesis would encourage more aggressive efforts elsewhere, such as the derivation of β-cells from human embryonic stem cells.

Genetic lineage tracing techniques have transformed our understanding of pancreas developmental biology, providing insights either unavailable from or contradictory to prior studies of fixed tissue [2]. For example, using the Cre-loxP system to monitor the fate of cells expressing the acinar protein *Carboxypeptidase A1 *(*Cpa1*) has revealed that these cells comprise a self-renewing, multipotent progenitor pool during mid-embryogenesis [7]. At later stages, *Cpa1*^+ ^cells become restricted to the acinar compartment, and lineage tracing of adult acinar cells indicates that they do not contribute to β-cells [7,8]. Other lineage tracing studies similarly cast doubt on the neogenesis model; for example, it is now appreciated that all islet cell types arise from *Neurog3*^+ ^precursor cells [9], yet *Neurog3*^+ ^cells are usually not detected after birth [10-12]. Similarly, a key study of adult β-cell expansion and self-renewal suggests that these processes reflect division of existing β-cells rather than neogenesis [13], a conclusion reinforced by subsequent independent findings [5,14].

Nonetheless, without a Cre line capable of marking duct cells, these studies could not exclude a minor ductal contribution to β-cells. Furthermore, they have not addressed the rapid expansion of β-cell mass that occurs shortly after birth [15], which may derive in part from neogenesis [6]. The ideal tool to address these questions would be a mouse line in which ducts can be inducibly marked at a specific time point, and their ability to contribute to β-cells determined at later stages: a cellular "pulse-chase" experiment [13]. Such mice could also clarify the origin of new β-cells formed in regeneration models, such as duct ligation and partial pancreatectomy, which may involve neogenesis [16,17]

Four transgenic mouse lines have recently been described in studies aimed at addressing this issue. Inada et al. [18] generated mice in which either Cre or its tamoxifen (TM)-inducible derivative CreER™ is driven by the promoter of *Carbonic anhydrase II *(*CAII*), a marker of adult duct cells. This study concluded that ducts continue to give rise to islets after birth, and that neogenesis is dramatically increased after duct ligation [18]. Means et al. [19] used a knock-in approach to target a similar tamoxifen-inducible CreERT molecule to the duct-specific *cytokeratin-19 *(*K19*) gene. *K19*^*CreERT *^labels many duct cells in neonatal and adult mice, as well as a small fraction of islet cells due to "leaky" recombinase expression in islets themselves. When *K19*^*CreERT *^was activated by TM treatment at birth, islet labeling at one week of age was no greater than expected from this background activity, suggesting that new islet cells generated in the interim did not derive from the more robustly-labeled duct population [19]. Finally, Solar et al. [20] generated a BAC transgenic in which TM-dependent CreERT2 is targeted to the first exon of *Hnf1β*, a transcription factor expressed selectively in embryonic and adult duct cells. This study indicated that *Hnf1β*^+ ^duct cells give rise to β-cells prior to birth but not thereafter, even in the context of injury models such as duct ligation [20]. The apparent contradiction between these studies, in particular those using *CAII- *and *Hnf1β*-driven Cre transgenes, suggests that additional markers should be sought out and exploited for their capacity to label duct cells, thereby providing independent evidence for or against postnatal neogenesis [21].

The gene *mucin1, transmembrane *(*Muc1*) is expressed throughout the ductal network in both the embryonic and adult pancreas, and excluded from islets [22,23]. We have generated mice in which the *Muc1 *gene is tagged with an *IRES-CreERT2 *cassette, permitting the inducible labeling of *Muc1*^+ ^cells. We find that this line also marks embryonic and postnatal acinar cells, reflecting endogenous Muc1 expression, but is completely excluded from islets in short-term "pulse" experiments. In the embryo, *Muc1*^+ ^cells give rise to endocrine cells as well as their *Neurog3*^+^precursors, formally confirming that islets originate from fetal ducts. Following birth, however, we find that the *Muc1*^+ ^lineage completely fails to contribute to new islet α- or β-cells, indicating that both the expansion and homeostasis of these cell types occurs independent of contribution from exocrine ducts or acini. These results argue against a major role for neogenesis in the normal postnatal pancreas, and set the groundwork for studies of potential neogenesis during regeneration.

## Results

### Targeting CreERT2 to the *Muc1 *locus

**Figure 1:**
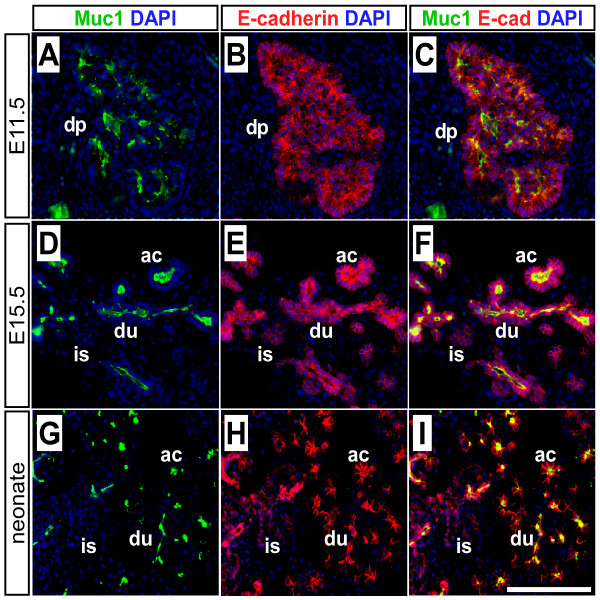
**Muc1 expression during pancreas development**. Double-immunofluorescence was performed to detect Muc1 (green) and the pan-epithelial marker E-cadherin (red) in sections of embryonic and neonatal pancreata. Muc1 is already strongly expressed in the E11.5 pancreatic epithelium (A-C), when most cells are still undifferentiated progenitors. From E15.5 (D-F) through birth (G-I) Muc1 expression remains strong and becomes restricted to cells lining ductal (du) and acinar (ac) lumens. Islet cells (is) do not express Muc1 at any stage. Scale bar: 100 μm. Abbreviations: ac, acinus; du, duct; is, islet.

**Figure 2:**
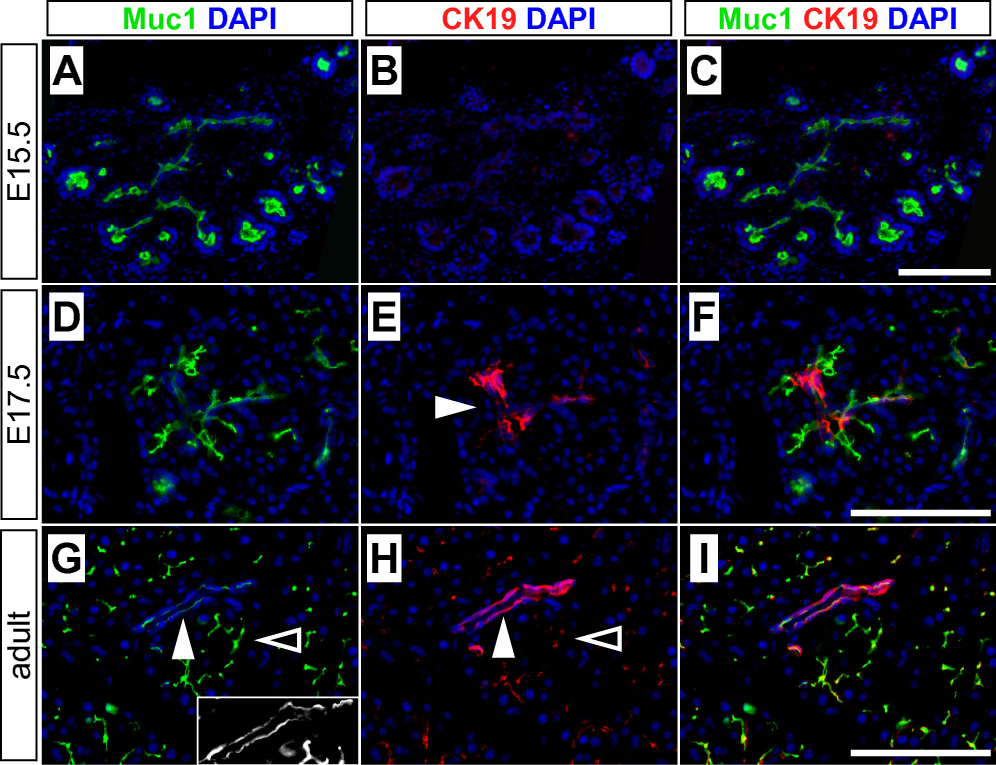
**Comparison of Muc1 and CK19 staining**. Double-immunofluorescence was performed to detect Muc1 (green) and cytokeratin-19 (CK19, red) in sections of embryonic and adult pancreata. (A-C) At E15.5, CK19 is undetectable while Muc1 is strongly expressed throughout the pancreatic ductal epithelium. (D-F) CK19 expression is first detected in larger ducts at E17.5, but remains weak or undetectable in smaller ones, while ducts of all sizes express Muc1. (G-I) In the adult pancreas, Muc1 protein is detected in all ducts, albeit at slightly lower levels in intralobular (closed arrowhead) than intercalated ducts (open arrowhead). (Insert in G demonstrates that Muc1 is expressed by all duct cells.) CK19 exhibits the opposite staining pattern, highest in intralobular and lowest in intercalated ducts. Scale bars: 100 μm.

To determine if the *Muc1 *locus could be exploited to mark ducts, we evaluated its expression in the embryonic and adult pancreas. We find that Muc1 is widely expressed at E11.5, when most pancreatic cells are still undifferentiated progenitors (Figure 1A-C). As embryogenesis proceeds, Muc1 expression persists in the branching epithelial network (Figure 1D-F), and it is expressed by all ductal cells of the mature organ (Figure 1G-I). Another duct marker, cytokeratin-19 (CK19), is undetectable prior to E17.5 (Figure 2). Interestingly, Muc1 expression appears to decrease as duct caliber increases, opposite to CK19 (Figure 2G-I). Nonetheless, we have not observed, at any stage, a Muc1-negative cell incorporated within a duct structure.

**Figure 3:**
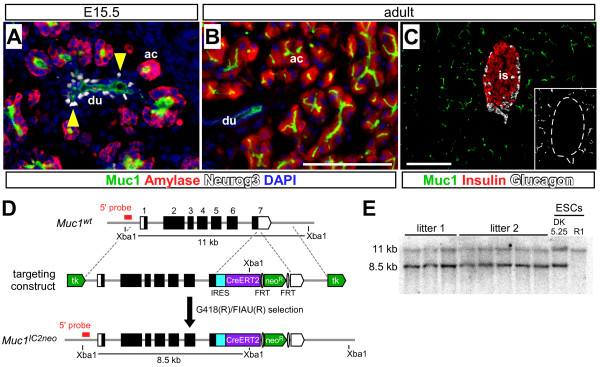
**Muc1 expression and gene targeting**. (A) At E15.5, Muc1 (green) is expressed by the ductal core, in which Neurog3^+ ^islet precursors (white, indicated by yellow arrowheads) appear to reside, as well as within the terminal elements of the epithelial network, adjacent to amylase-expressing acini (red). (B) While Muc1 remains strongly expressed throughout the adult ductal tree, Neurog3 is not detectable. (C) Muc1 (green) is not detected within or adjacent to adult islet β-cells (insulin, red) or α-cells (glucagon, white). Inset depicts Muc1 staining alone (white), with islet boundaries indicated by dashed line. (D) Structure of *Muc1 *locus, targeting vector and targeted allele. The targeting vector was designed to introduce an *IRES-CreERT2 *cassette downstream of the Muc1 stop codon, along with a FRT-flanked *neo*^*R *^construct for positive G418 selection and a *tk *gene at the end of each homology arm for negative FIAU selection in ES cells. Dotted lines indicate the boundaries of the 5' and 3' homology arms used for targeting, and the probe used for Southern blotting (outside the 5' homology arm) is indicated in red. Correct targeting introduces a new XbaI site, shifting the predicted restriction fragments detected by this probe. (E) Southern blotting of *Muc1*^*IC*2*neo *^ES cells and mice. Xba1 digests of genomic DNA prepared from ES cells (parental R1 cells or targeted clone DK5.25), as well as from putative *Muc1*^*IC*2*neo*/+ ^F1 offspring of DK5.25 chimeras (genotyped by PCR), were hybridized with the probe indicated in panel A. Targeted ES cells and mice display both wildtype (11 kb) and *IC2neo *(8.5 kb) XbaI fragments. Scale bars: 100 μm. Abbreviations: ac, acinus; du, duct; is, islet.

Embryonic islet precursors express the transcription factor *Neurogenin3 *(*Neurog3*), and are considered to arise from primitive duct-like cells [4,9-11]. We find that Neurog3 expression localizes within or in close proximity to Muc1^+ ^cells at E15.5, consistent with a ductal origin for islet cells (Figure 3A). While Neurog3 expression is not detectable in adults, Muc1 remains expressed throughout the ductal network of the pancreas, including large ducts as well as fine terminal branches within acini (Figure 3B). Importantly, Muc1 expression is excluded from islet cells during embryogenesis as well as in the mature organ (Figs. 1, 3C). Altogether, *Muc1 *appears to satisfy our requirements for a Cre driver line to study islet neogenesis: it is expressed in all duct cells, embryonic and adult, and excluded from differentiated islets.

As detailed in the Methods, we generated a *Muc1*^*IRES*-*CreERT*2 ^allele (henceforth, *Muc1*^*IC*2^) by gene targeting, introducing an *IRES-CreERT2 *cassette after the endogenous stop codon of the Muc1 locus (Figure 3D-E). The CreERT2 protein is a tamoxifen (TM)-dependent recombinase that provides temporal control of Cre activity [24], which should allow us to investigate the differentiation potential of embryonic and postnatal duct cells. *Muc1*^*IC*2 ^heterozygous and homozygous mice are viable and fertile (data not shown).

### *Muc1*^*IC2 *^marks exocrine cells in the adult pancreas

**Figure 4:**
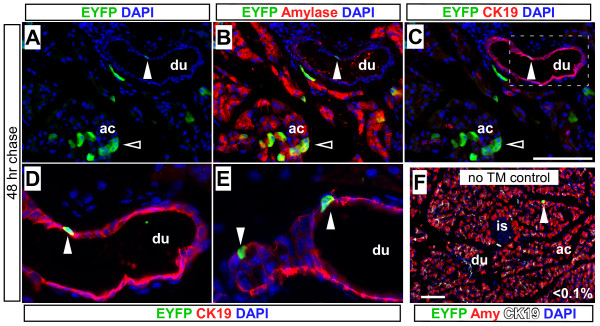
***Muc1*^*IRES*-*CreERT*2 ^is active in adult exocrine cells**. (A-C) Recombination was induced in adult (P60) *Muc1*^*IC*2/+^;*Rosa26*^*EYFP*/+ ^mice by administration of 10 mg tamoxifen, and mice were analyzed 2 days later by immunofluorescence for EYFP (green), amylase (B, red) and cytokeratin-19 (C, red), together with DAPI (blue) to mark nuclei. After a short term chase, *Muc1*^*IC*2 ^marks cells of the ductal network (closed arrowheads) as well as acinar cells (open arrowheads), suggesting that Muc1 is expressed in both duct and acinar cells. (D-E) Ductal *Rosa26*^*EYFP *^labeling by *Muc1*^*IC*2^, shown in fields devoid of labeled acinar cells (D, magnified from panel C; E, independent field). As *Rosa26*^*EYFP *^expression is lower in duct cells than acini, we have increased the green signal in these panels to make ductal staining more obvious. (F) 8-month old TM-untreated control mouse, stained for EYFP (green), amylase (red) and cytokeratin-19 (white). Less than 0.1% of duct and acinar cells are labeled in the absence of TM. Scale bars: 100 μm. Abbreviations: ac, acinus; du, duct; is, islet.

To determine whether *Muc1*^*IC*2 ^marks ducts, we performed short-term labeling experiments with the Cre-dependent EYFP reporter strain, *Rosa26*^*EYFP *^[25]. We induced recombination by treating adult (6-8 week-old, n = 3) *Muc1*^*IC*2/+^;*Rosa26EYFP*^/+ ^mice with a single dose of 10 mg tamoxifen. After a two-day "chase" period, we found the EYFP lineage label not only in cytokeratin-19^+ ^duct cells (~6%), as expected, but also within amylase^+ ^acinar cells (Figure 4A-E, ~12%), indicating that this Cre line is active in both duct and acinar cells. (Note that in this and other experiments, we find that *Rosa26*^*EYFP *^appears to drive stronger EYFP expression in acinar cells than ducts.) An identical labeling distribution was observed using a different reporter strain, *Rosa26*^*LacZ *^[26] (data not shown). In these and other experiments, all *Muc1*^*IC*2^-labeled cells were found to be E-cadherin^+ ^(data not shown), indicating that they represent pancreatic parenchymal cells rather than connective tissue or vasculature.

**Figure 5:**
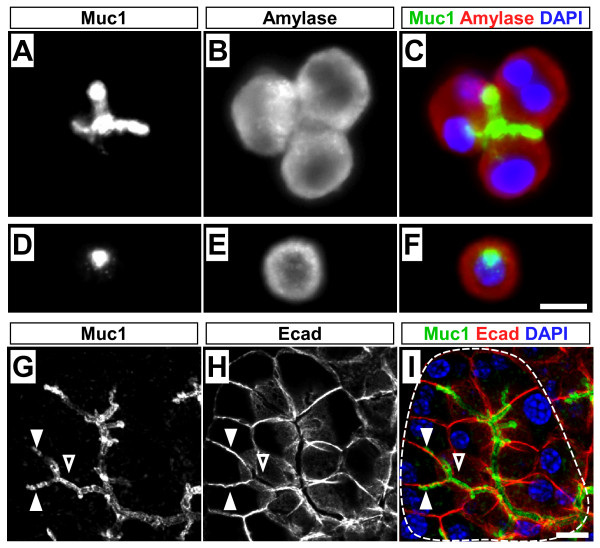
**Muc1 localization within acinar structures**. (A-F) Immunofluorescence of dissociated wildtype acini for Muc1 (green), amylase (red) and DAPI (blue). Muc1 is detected within small clusters of amylase^+ ^acinar cells as well as in single amylase^+ ^cells, suggesting that these cells, as well as ducts, express the *Muc1 *gene. (G-I) Confocal z-projection (0.75 μm) of adult wildtype pancreas, wholemount stained for Muc1 (green), E-cadherin (red) and DAPI (blue). Outline in I indicates a single acinar unit, with a shared central lumen. Membrane-bound Muc1 protein localizes to this central lumen (open arrowhead), as well as extending between two individual acinar cells (closed arrowhead). Note that there appears to be no centroacinar cell in this cluster, the lumen of which is entirely defined by the apical surfaces of adjacent acinar cells, and coated by Muc1 protein. Scale bars: 10 μm.

Having observed the lineage label in acinar cells, we wanted to confirm that these cells actually express Muc1. After enzymatic dissociation and immunostaining of isolated acini, we detect Muc1 not only within small acini, comprised entirely of amylase^+ ^cells, but also in single amylase^+ ^cells (Figure 5A-F). Confocal imaging of wholemount stained pancreata confirmed that Muc1 protein localizes to the apical poles of acinar cells, and is readily detected within individual acini that lack centroacinar cells [27] (Figure 5G-I). Since this antibody recognizes the intracellular domain of the Muc1 protein [28], the observed staining is unlikely to reflect shed or contaminating Muc1 from duct or centroacinar cells. In situ hybridization studies have similarly revealed *MUC1 *expression in both acinar and duct cells of the human pancreas [29], and we conclude that the acinar labeling observed with *Muc1*^*IC*2 ^reflects endogenous *Muc1 *expression.

**Figure 6:**
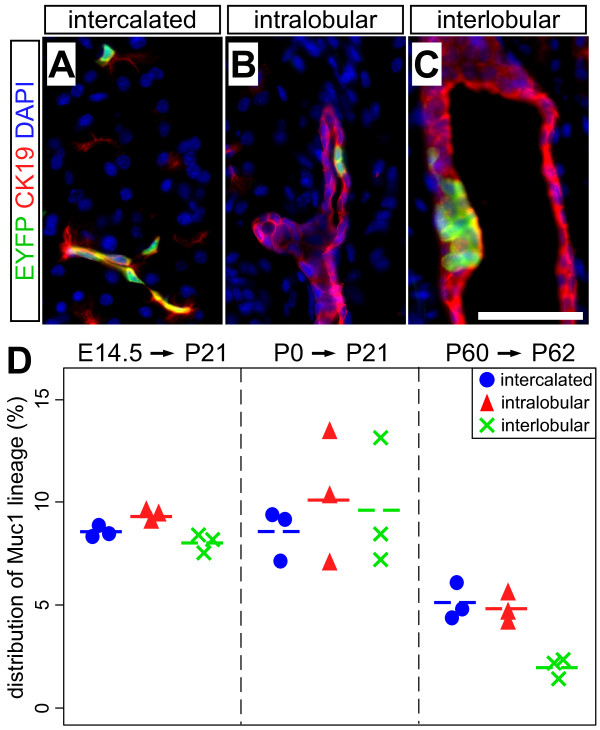
***Muc1*^*IC*2 ^labels cells throughout the ductal network**. (A-C) Representative immunofluorescence images documenting the presence of *Muc1*^*IC*2^-labeled cells (green) within small/intercalated, medium/intralobular and large/interlobular ducts, co-stained for cytokeratin-19 (red). Interlobular were distinguished from intralobular ducts by the presence of connective tissue separating them from acinar parenchyma. Scale bar: 50 μm. (D) *Muc1*^*IC*2 ^lineage-labeling indices of different duct populations, within various pulse-chase experiments. In each experiment, we observe roughly equal labeling of intercalated (blue circle), intralobular (red triangle) and interlobular (green X) ducts, with the exception of decreased intercalated duct labeling after tamoxifen treatment of adults (p < 0.01 by TukeyHSD).

We also determined the relative distribution of *Muc1*^*IC*2^-labeled cells between small (intercalated), medium (intralobular) and large (interlobular) ducts (Figure 6A-C). We found that *Muc1*^*IC*2^-labeled cells were present in all three duct types, with slightly lower labeling within interlobular ducts than those of smaller caliber (Figure 6D). This labeling distribution is consistent with the distribution of Muc1 protein itself, described above (Figure 2), and suggests that *Muc1*^*IC*2^-driven recombination can occur in any *Muc1*-expressing cell.

Examining TM-untreated 4-8 month *Muc1*^*IC*2/+^;*Rosa26EYFP*^/+ ^and *Muc1*^*IC*2/+^;*Rosa26LacZ*^/+ ^mice (n = 3) revealed a background recombination rate of less than 0.1% (Figure 4F and data not shown), confirming that this line exhibits stringent tamoxifen dependence. Furthermore, we never observed lineage-labeled endocrine cells in our short-term experiments. *Muc1*^*IC*2 ^labeling is thus TM-dependent, as expected, and extends throughout the exocrine pancreas (acini and ducts). Although TM-induced *Muc1*^*IC*2 ^labeling is relatively sparse (5-15% of exocrine cells, in this experiment), its distribution closely matches that of Muc1 itself, and appears to represent a random sampling of the exocrine pancreas. As *Muc1*^*IC*2 ^does not directly label islet cells, it can be used to detect potential exocrine-derived neogenesis.

### Islet cells arise from embryonic *Muc1*^+ ^cells

**Figure 7:**
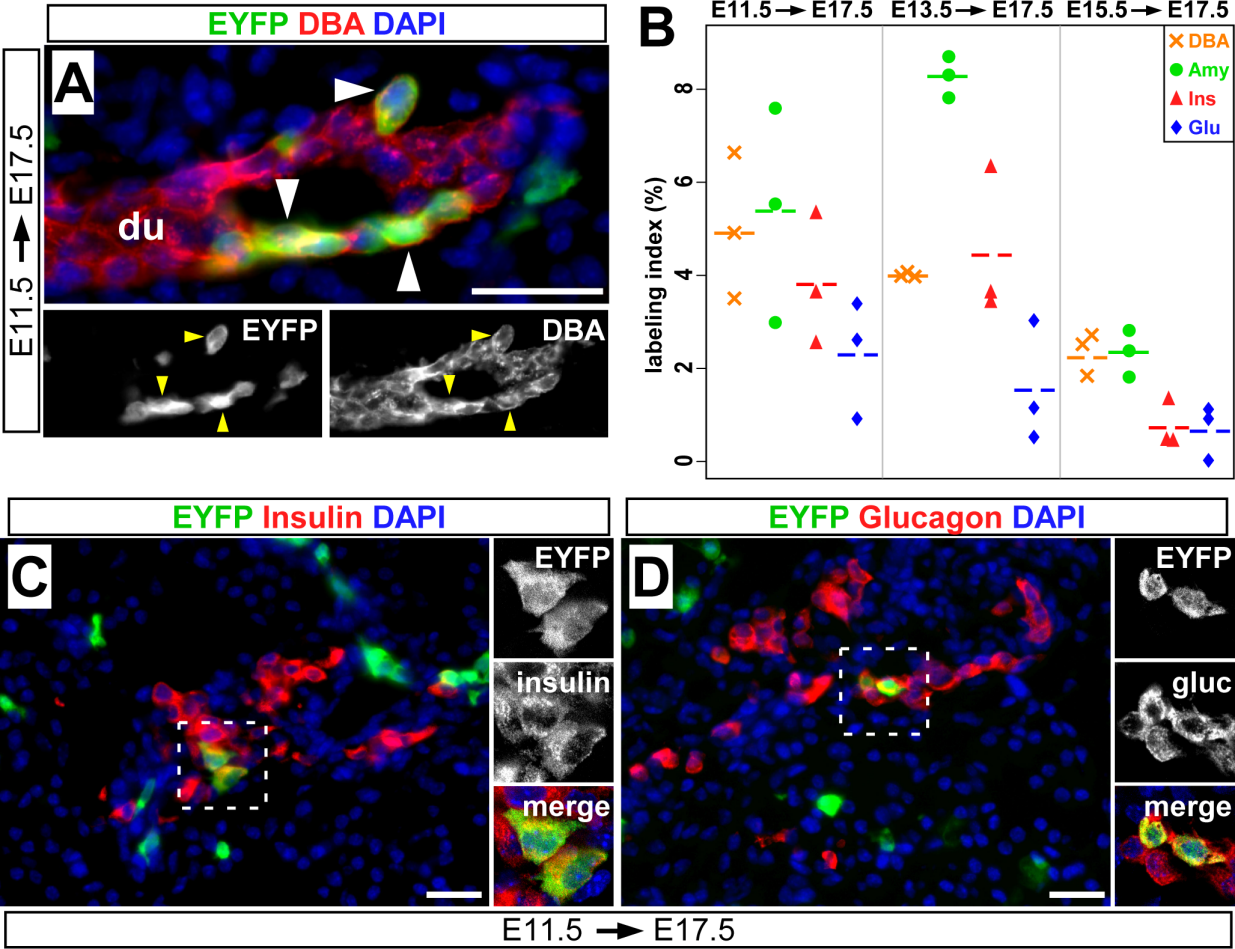
***Muc1*^+ ^cells give rise to endocrine cells during embryogenesis**. (A) A single dose of 5 mg tamoxifen was given to pregnant mice at E11.5, and pancreata of *Muc1*^*IC*2/*IC*2^; *Rosa26*^*EYFP*/+ ^offspring were analyzed at E17.5 by immunofluorescence for EYFP (green), DBA lectin (red) and DAPI (blue). Arrowheads indicate DBA^+ ^cells co-expressing EYFP. (B) Quantification of observed co-labeling at E17.5, following tamoxifen administration at indicated time points. Scatter plot depicts the percentages of DBA^+ ^(orange), amylase^+ ^(green), insulin^+ ^(red) and glucagon^+ ^(blue) cells co-expressing EYFP. Each point represents an independent embryo, and means are indicated by dotted lines. (C-D) Pancreata of *Muc1*^*IC*2/*IC*2^; *Rosa26*^*EYFP*/+ ^embryos, administered TM at E11.5 as in (A), stained at E17.5 for EYFP (green), insulin or glucagon (red) and DAPI (blue). Inserts: confocal z-projection (1.36 μm) of the boxed areas, to confirm localization of EYFP to hormone-producing cells. Scale bars: 25 μm.

To determine whether *Muc1*^+ ^cells contribute to islet neogenesis in utero, we administered tamoxifen to pregnant females carrying *Muc1*^*IC*2/+^; *Rosa26EYFP*^/+ ^embryos. These experiments yielded a very low overall labeling rate (≤1% of any cell type, data not shown), possibly due to reduced amounts of TM entering fetal circulation. To increase the cellular Cre concentration, therefore, we used homozygous *Muc1*^*IC*2/*IC*2^; *Rosa26EYFP*^/+ ^embryos for all in utero labeling experiments. *Muc1*^*IC*2/*IC*2^;*Rosa26EYFP*^/+ ^embryos were labeled with a single maternal dose of TM (5-10 mg) at different embryonic stages: E11.5, E13.5 or E15.5. All embryos were analyzed at E17.5 (n = 3 for each tamoxifen treatment group). As expected, *Muc1*^*IC*2 ^labels fetal ducts at all these timepoints (Figure 7A and data not shown), albeit at low frequencies (2-6%, Figure 7B). Consistent with its labeling pattern in the adult pancreas, *Muc1*^*IC*2 ^marks a similar proportion of fetal acinar cells as well (Figure 3B and data not shown). Furthermore, we observe EYFP lineage-labeling of insulin^+ ^β-cells and glucagon^+ ^α-cells in all of these pancreata (Figure 7B-D), indicating an origin within the *Muc1*^+ ^exocrine compartment. Our experimental design therefore identifies potential neogenesis even from a sparsely-labeled population. As β-cell numbers increase exponentially between E11.5 and E17.5 [30], ~100-fold total, most of the β-cells analyzed in this experiment would have been born since tamoxifen was administered. That we find comparable *Muc1*^*IC*2 ^lineage labeling in β-cells and exocrine cells (Figure 7B) suggests that most of the new β-cells arose via exocrine-derived neogenesis.

**Figure 8:**
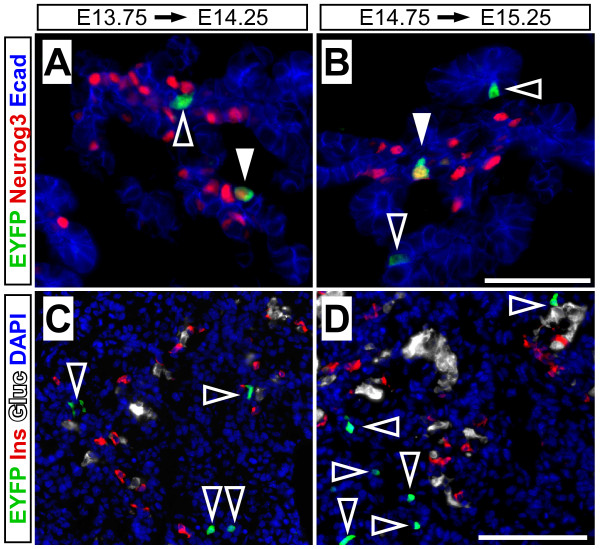
**Neurog3^+ ^cells arise from *Muc1*^+ ^ductal cells during embryogenesis**. To detect the short-term differentiation potential of *Muc1*^+ ^cells, *Muc1*^*IC*2/*IC*2^; *Rosa26EYFP*^/+ ^embryos were labeled by TM administration to pregnant dams at E13.75 (A, C) or E14.75 (B, D), and harvested ~12 hours later for immunofluorescence analysis (n = 2 for each timepoint). (A-B) Short-term labeling of *Muc1*^+ ^cells (green) reveals their presence within the E-cadherin^+ ^ductal network (blue, open arrowheads), as well as their contribution to Neurog3^+ ^cells (red, closed arrowheads). (C-D) Similar analysis of islet hormone expression indicates that *Muc1*^*IC*2 ^labeling (green, open arrowheads) is excluded from insulin^+ ^(red) and glucagon^+ ^cells (white), confirming that these cells derive from *Muc1*^+ ^progenitors but do not themselves express *Muc1*. Scale bars: 50 μm in A-B and 100 μm in C-D.

To further confirm that *Muc1*^*IC*2 ^is not active in fetal islets themselves, we performed short-term (12-14 hr) pulse-chase labeling of embryos at E13.75 or E14.75. We could easily detect the lineage label in the embryonic ductal system as well as in Neuog3^+ ^cells (Figure 8A-B), but failed to detect any labeled hormone-producing cells (Figure 8C-D). The short-term labeling of Neurog3^+ ^cells agrees with the co-expression of Neurog3 and Muc1 in embryonic ducts (Figure 3A), and the lack of short-term islet labeling confirms that the later appearance of labeled endocrine cells reflects differentiation from exocrine tissue.

**Figure 9:**
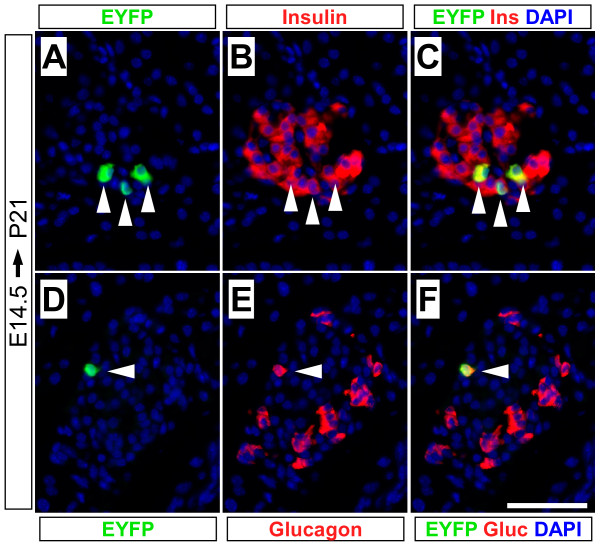
**Postnatal islet cells derive from embryonic *Muc1*^+ ^progenitors**. *Muc1*^*IC*2/*IC*2^; *Rosa26*^*EYFP*/+ ^mice received tamoxifen in utero (5 mg to pregnant dam at E14.5), and were analyzed at P21 by immunofluorescence for EYFP lineage label (green), insulin (A-C, red) and glucagon (D-F, red). After inducing the Muc1 lineage at E14.5 and chasing offspring until P21, EYFP labeling (white arrowheads) is detected among both insulin^+ ^β-cells (2.2 +/- 0.4%) and glucagon^+ ^α-cells (2.1 +/- 0.2%). Scale bar: 50 μm.

Finally, to determine whether fetal *Muc1*^+ ^cells contribute to adult islets, we administered 5 mg TM to pregnant females at E14.5 and analyzed labeling at weaning age (P21). We found labeling of both β-cells (2.2 +/- 0.4%) and α-cells (2.1% +/- 0.2%), present in mature islet structures (Figure 9). As expected, lineage label also persists in adult duct cells, and appears to be distributed equally among various classes of duct structures (Figure 6D).

Altogether, these results strongly suggest that (a) embryonic *Muc1*^+ ^duct cells give rise to all segments of the adult ductal network, (b) *Muc1 *is not expressed by endocrine cells and (c) mature islet cells arise from *Neurog3*^+ ^precursors within the embryonic *Muc1*^+ ^ductal network. These results represent direct evidence that endocrine cells arise from embryonic ducts, but they leave open the possibility that embryonic *Muc1*^*IC*2 ^labeling actually marks a ductal stem cell-like population, which continues to give rise to new islet cells after birth. We therefore turned our attention to the differentiation potential of postnatal *Muc1*^+ ^cells.

### *Muc1*^+ ^exocrine cells do not undergo endocrine differentiation after birth

**Figure 10:**
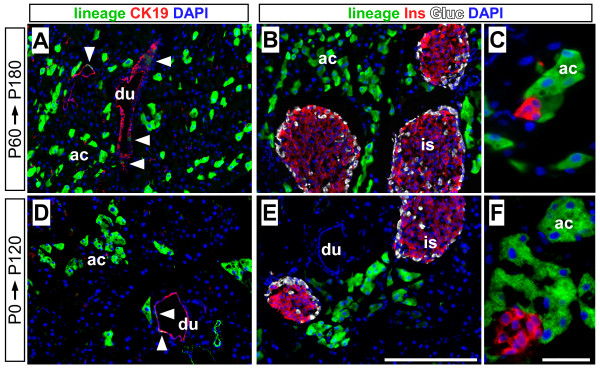
**The *Muc1 *lineage does not contribute to islets after birth**. (A-C) Experiment 1: adult *Muc1*^*IC*2/+^; *Rosa26*^*EYFP*/+ ^mice (P60) were treated with a single dose of 10 mg tamoxifen, and analyzed after a 120 d chase period (i.e. to six months of age). Immunofluorescence was performed for EYFP lineage label (green), cytokeratin-19 (A, red), insulin (B-C, red) and glucagon (B, white). Lineage label remains restricted to the exocrine compartment (arrowheads indicate labeled duct cells). (D-F) Experiment 2: *Muc1*^*IC*2/+^; * Rosa26*^*LacZ*/+ ^neonates received TM via maternal gavage (sequential 10 mg doses at P0 and P1), and were analyzed after 120 d by immunofluorescence as per A-C (using LacZ, in green, as lineage label). LacZ labeling is confined to duct (white arrowheads) and acinar cells, with no detectable expression in islet cells. Scale bars: A-B and D-E, 100 μm; C and F, 25 μm. Abbreviations: du, duct cells; ac, acini.

We performed three experiments to determine whether the *Muc1*^+ ^exocrine compartment contributes to islet cells after birth. In experiment 1, we administered 10 mg tamoxifen to young adult (~P60) *Muc1*^*IC*2/+^; *Rosa26EYFP*^/+ ^mice, and assayed the potential contribution of labeled cells to either exocrine or endocrine cells after 7, 60 or 120 days (Figure 10A-C and data not shown). We observed an overall labeling efficiency (i.e. proportion of EYFP^+ ^cells per field, without reference to cell type-specific markers) of ~24% in these experiments, which included ~30% labeling of acinar and ~10% labeling of duct cells (Table 1). As our short-term labeling experiments never revealed detectable lineage marking of islet cells (D.K., unpublished observations), we focused our quantitative analyses on the 60 d and 120 d chase periods. We counted randomly-chosen fields of β-cells and α-cells, scoring separately for number of insulin^+ ^or glucagon^+ ^cells, number of EYFP^+ ^cells, and number of double-positive (hormone^+^/EYFP^+^) cells. In fact, after scoring several thousand cells positive for each marker (Table 2), we never observed a single β-cell or α-cell positive for EYFP, suggesting that new islet cells do not arise in significant numbers from adult *Muc1*^+ ^exocrine cells.

**Table 1:** *Muc1*^*IC*2 ^lineage contribution to exocrine cells

					**Lineage**^**+ **^**(EYFP or LacZ)**		
					
Experiment	Genotype	Pulse protocol	Chase period	Sample #	**% of DAPI**^**+**^	**% of AMY**^**+**^	**% of CK19**^**+**^
**1**	**Muc1**^**IC2/+**^**; Rosa26**^**EYFP/+**^	**adult (P60)** 10 mg TM gavage	**7 d**	1	23.6	29.3	7.5
				2	22	27.3	6.2
				3	24.3	32.2	5.4
				**mean**	**23.3 ± 0.7**	**29.6 ± 1.4**	**6.4 ± 0.6**
			
			**120 d**	1	30	42.4	8.5
				2	12.6	15	11.7
				3	20.8	26.4	10.1
				4	33	43.6	14.5
				5	23.6	30.6	13.3
				**mean**	**24.0 ± 3.6**	**31.6 ± 5.3**	**11.6 ± 1.1**
			
			**overall mean**		**23.7 ± 2.2**	**30.8 ± 3.2**	**9.6 ± 1.2**

**2**	**Muc1**^**IC2/+**^**; Rosa26**^**LacZ/+**^	**pups** **(P0/P1)** 10 mg TM maternal gavage	**21 d**	1	16.6	22.9	9.9
				2	11.7	15.5	3.6
				3	3.3	4.1	1.8
				4	6.2	7.4	1.9
				5	5.1	5.9	1.5
				**mean**	**8.6 ± 2.4**	**11.2 ± 3.5**	**3.7 ± 1.6**
			
			**120 d**	1	14.1	21.9	5.5
				2	21.4	30.9	6.2
				**mean**	**17.7 ± 3.7**	**26.4 ± 4.5**	**5.8 ± 0.4**
			
			**overall mean**		**10.6 ± 2.5**	**15.5 ± 3.8**	**4.3 ± 1.2**

**3**	**Muc1**^**IC2/+**^**; Rosa26**^**EYFP/+**^	**pups (P0)** 2 mg TM SQ	**21 d**	1	21.7	ND	8.9
				2	22.8		7.8
				3	51.1		13.5
				**mean**	**31.9 ± 9.6**		**10.1 ± 1.7**

Three "pulse-chase" lineage-labeling experiments were performed, as described in the text, in which tamoxifen was administered (by oral gavage to adults, or subcutaneous injection to neonates) and mice sacrificed after chase periods of 7-120 days. For each sample, we counted total cells per field (DAPI), acinar cells (amylase^+^) and duct cells (cytokeratin-19^+^), as well as the number of cells double-positive for these markers and for the lineage tracer. From these counts, we derived the labeling efficiency (expressed as a percentage) of total cells (DAPI), acinar (AMY) and duct (CK19) cells. Shown are summary data for every mouse analyzed, as well as mean values +/- standard error. ND, not determined.

**Table 2:** Quantification of potential Muc1 contribution to endocrine cells

					number of cells scored		
					
Experiment	Genotype	Pulse protocol	Chase period	Sample #	**lineage**^**+**^	**INS**^**+**^	**GLU**^**+**^
**1**	**Muc1**^**IC2/+**^**; Rosa26**^**EYFP/+**^	**adult (P60)** 10 mg TM gavage	**60 d**	1	2950	2054	ND
				2	959	1398	
				**total**	**3909**	**3452**	
			
			**120 d**	1	1588	624	209
				2	1416	498	144
				3	1266	1069	374
				4	2089	2004	703
				5	1199	1488	409
				6	1079	904	369
				**total**	**8637**	**6587**	**2208**

**2**	**Muc1**^**IC2/+**^**; Rosa26**^**LacZ/+**^	**pups** **(P0/P1)** 10 mg TM maternal gavage	**21 d**	1	221	590	276
				2	483	745	235
				3	266	1045	436
				**total**	**970**	**2380**	**947**
			
			**120 d**	1	2072	2152	626
				2	2637	1847	315
				**total**	**4709**	**3999**	**941**

**3**	**Muc1**^**IC2/+**^**; Rosa26**^**EYFP/+**^	**pups (P0)** 2 mg TM SQ	**21 d**	1	1566	2017	999
				2	1264	950	488
				3	3508	1570	564
				**Total**	**6338**	**4537**	**2051**

As described in the text, mice subjected to "pulse-chase" labeling with *Muc1*^*IC*2 ^were analyzed for potential contribution of lineage-marked cells to insulin^+ ^β-cells or glucagon^+ ^α-cells (exocrine labeling results in Table 1). Indicated are the total number of cells scored as positive for lineage label (EYFP or LacZ), as well as the total number of insulin^+ ^and glucagon^+ ^cells scored in the same fields. In no case did we observe a lineage label-positive endocrine cell. ND, not determined.

To determine if *Muc1*^+ ^cells contribute to the rapid expansion of islet cell numbers after birth [15], we performed experiment 2, in which we induced recombination in *Muc1*^*IC*2/+^; *Rosa26*^*LacZ*/+ ^neonates by administration of tamoxifen to nursing mothers (consecutive 10 mg doses on P0 and P1). We sacrificed mice 21 or 120 days after treatment (i.e. as weanlings or mature adults), and performed immunofluorescence to detect LacZ lineage marker within the exocrine and endocrine compartments (Figure 10D-F and data not shown). We found an overall labeling efficiency of ~10% in these experiments, including ~15% of acinar cells and ~4% of duct cells (Table 1). As in experiment 1, however, despite scoring several thousand cells for each marker, we did not observe a single LacZ^+ ^β-cell or α-cell (Table 2).

Previous studies indicate that acinar cells do not contribute to islets after birth [7,8,31], and experiment 2 suggests that neonatal duct cells are also excluded from the islet lineage. This interpretation hinges on relatively infrequent ductal labeling, which could have hidden a low level of duct-derived neogenesis. To increase the duct labeling frequency, we performed experiment 3, in which we directly injected newborn *Muc1*^*IC*2/+^; * Rosa26EYFP*^/+ ^pups with tamoxifen (2 mg per pup, delivered subcutaneously). Upon sacrifice, 21 days after TM administration, we found increased overall labeling compared to mice that received maternal TM (~30% EYFP^+^, Table 1). Importantly, the duct labeling frequency was increased to 10%, with equal distribution among interlobular, intralobular and intercalated ducts (Figure 6D). Nonetheless, we did not observe any labeled β-cells or α-cells, despite scoring several thousand cells positive for each marker (Table 2).

These analyses suggest an upper limit to the contribution of neogenesis to postnatal islet growth. β-cell mass has been reported to expand between 4- and 10-fold in the first 2-4 weeks after birth [32-34]. If we assume a five-fold expansion between P0 and P21, we can infer that ~80% of the β-cells scored in experiment 3 were "new" since P0 (3600 of the ~4500 β-cells counted, Table 2). If all of these had been derived from *Muc1*^*IC*2^-labeled duct cells, given a duct labeling index of ~10% (Table 1), we would have expected to observe roughly 360 labeled β-cells. As we observed zero, we conclude that ≤1% of all β-cells generated after birth could have arisen from labeled ducts (1% neogenesis would have resulted in ~4 labeled β-cells, which is probably near the limit of reliable detection). Altogether, experiments 1-3 fail to reveal duct-to-islet transdifferentiation after birth.

## Discussion

**Figure 11:**
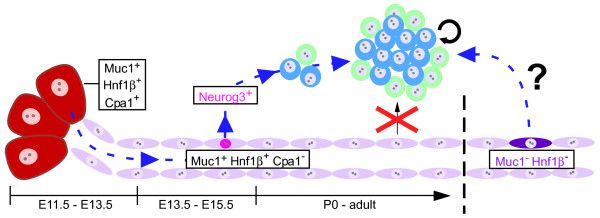
**Dynamic differentiation potential within theexocrine pancreas**. Multipotent pancreatic progenitors (E11.5-E13.5) express the digestive enzyme Cpa1, which is later restricted to acinar cells (E14.5-adult), together with Muc1 and Hnf1β [20]. While *Cpa1*^+ ^cells cease contribution to islets from approximately E13.5 [7], *Muc1*^+^/*Hnf1β*^+ ^cells continue to give rise to *Neurog3*^+ ^endocrine precursors (pink nuclei) and islets (blue and green) through at least E15.5. From around birth, *Muc1*^+ ^and *Hnf1β*^+ ^cells no longer contribute to the endocrine lineage, and mature islets are maintained by self-replication of those pre-existing. However, it remains a formal possibility that a subpopulation exists within the ductal network (dark blue cytoplasm), expressing neither *Muc1 *nor *Hnf1β*, from which β-cells continue to arise after birth.

At birth, the mammalian β-cell changes from a metabolic passenger to the driver of glucose homeostasis. Based on our results and those of Solar et al. [20], we propose that the mechanisms controlling β-cell mass also change at birth, from a fetal period of new differentiation, or neogenesis, to a mature state of self-renewal (Figure 11). To detect this transition, we performed a direct comparison of duct and acinar cell lineages before and after birth. We provide formal proof -- confirming prior studies of histology and gene expression -- that islets arise from embryonic *Muc1*^+ ^ducts. From birth onwards, however, we find no evidence for a ductal origin of new β-cells, and we propose that postnatal β-cell expansion and homeostasis normally occur without contribution from ducts or acini.

We had intended, in creating the *Muc1*^*IC*2 ^allele, to specifically address the differentiation potential of duct cells. Instead, we find that *Muc1*^*IC*2 ^labels both acinar and duct cells, at all stages examined, and that Muc1 protein is readily detected within acinar cells. Nonetheless, we can treat the labeling of postnatal acinar cells as "background," as acinar-to-islet transdifferentiation does not occur after birth [7,8,31]. Cells expressing the acinar enzyme *Cpa1 *do behave as multipotent "tip cell" progenitors prior to E13.5, but are thereafter restricted to the acinar lineage [7]. As *Muc1*^+ ^cells still contribute to islets at E13.5 and E15.5 (Figs. 7, 9), we propose that islet differentiation competence normally shifts from *Muc1*^+^/*Cpa1*^+ ^tips to *Muc1*^+^/*Cpa1*-negative "trunks" after E13.5, before being lost entirely at birth (Figure 11).

Another recently developed mouse line, *K19*^*CreERT*^, in which CreERT is targeted to the *cytokeratin-19 *locus, drives TM-dependent recombination in inter- and intralobular ducts [19]. Unlike *Muc1*^*IC*2^, *K19*^*CreERT *^does not label distal intercalated ducts, and is active in a small fraction of islet cells. Nonetheless, preliminary experiments reported using *K19*^*CreERT *^provide independent evidence supporting our model: TM treatment at birth results in ≥10% labeling of ducts after one week, but <1% labeling of islets, equivalent to the direct activity of this line in islet cells themselves [19].

While this manuscript was in preparation, Solar and colleagues [20] published a study using another exocrine CreERT2 line, driven by the *Hnf1β *locus. Unlike *Muc1*^*IC*2^, this driver is not active in acini, and labels a higher fraction of duct cells postnatally (approximately 20% at birth and 40% in adults, compared to 10% labeling at either timepoint with *Muc1*^*IC*2^). As with *Muc1*^*IC*2^, lineage-tracing of *Hnf1β*^+ ^cells revealed duct-to-islet differentiation prior to birth, but none thereafter. Further experiments by these investigators indicate that such differentiation does not occur in the context of injury and regeneration [20], as previously believed [16].  Our data provide further evidence against postnatal duct-to-islet differentiation in the healthy pancreas, although it remains to be determined if injury can induce neogenesis from *Muc1*^*IC*2^-expressing population.

The *Hnf1β-CreERT2 *and *Muc1*^*IC*2 ^lineage tracing results contradict those obtained with a Cre transgene driven by the *Carbonic anhydrase II *promoter (*CAII-Cre*) [18]. Using *Rosa26*^*LacZ *^reporter mice to detect recombination [26], these authors report that *CAII-Cre *drives duct-restricted recombination beginning at E18.5, but labels roughly 15% of β-cells at four weeks of age. We cannot offer an obvious explanation for this discrepancy; given the number of β-cells that we counted, we should have detected such a robust contribution from *Muc1*^*IC*2^-labeled duct cells. One possibility is that *CAII-Cre*-catalyzed recombination actually begins prior to birth, when *Neurog3*^+ ^cells are still present [10,11], but that LacZ expression cannot be detected until one or more days after birth. In fact, half of the newborn pancreata examined in this study already exhibited at least some labeled β-cells (in one pup, as many as 70% of islets contained labeled β-cells), indicating prenatal recombination [18]. These authors have also generated a *CAII-CreERT *transgene, which could be used to follow postnatal labeling specifically, although their experiments with these mice revealed surprisingly high levels of tamoxifen and Cre-independent LacZ expression in adult islets [18].

Alternatively, a subpopulation of duct cells with the capacity for islet differentiation might escape labeling by *Muc1*^*IC*2^, *K19*^*CreERT *^and *Hnf1β-CreERT2*, but not by *CAII-Cre *(Figure 11). Indeed, as neither our Cre driver nor those described by others labels a majority of postnatal duct cells [19,20], the possibility of a substantial unmarked subpopulation is impossible to exclude formally. As three independent and distinct Cre transgenes have yielded identical conclusions, however, the burden of evidence would appear to weigh against postnatal islet neogenesis.

With respect to *Muc1*^*IC*2 ^in particular, we note that although its recombination efficiency in utero is even lower than after birth, our experimental approach still identifies islet cells arising from the sparsely labeled embryonic exocrine compartment. Furthermore, we have never observed a Muc1-negative duct cell (Figs. 1, 2, 3, and data not shown), nor is there evidence for anatomical exclusion of *Muc1*^*IC*2^-labeled cells within the ductal network (Figure 6). We also do not observe any obvious change in Muc1 expression or distribution between embryonic stages, when *Muc1*^*IC*2 ^does label endocrine cells, and postnatal stages, when it does not. An obvious transition that does occur perinatally is the extinction of *Neurog3 *expression, which itself weighs against the persistence of duct-to-islet differentiation after birth [10,11].

From a physiological perspective, it might make sense that expansion of β-cells after birth involves a mechanism independent of neogenesis, as postnatal β-cells must contend with metabolic demands from which embryonic progenitor cells are buffered. In fact, numerous knockout mouse studies indicate the existence of postnatal-specific mechanisms to control β-cell mass [32,35-39]. Furthermore, recent studies suggest that expansion of β-cell mass in adults, in response to β-cell damage or increased insulin demand, occurs via proliferation rather than neogenesis [13,37,40]. Potential exceptions to this rule have been described, including partial pancreatectomy and duct ligation, in which development of new β-cells is accompanied by the re-appearance of *Neurog3*-expressing cells within the ductal epithelium [16,17]. Studies using *Hnf1β-CreERT2 *to mark pre-existing duct cells in such models did not detect contribution to new β-cells, however [20]. The *Muc1*^*IC*2 ^line is well suited for similar experiments and, as a tool to mark cells throughout the exocrine pancreas, it should complement and extend results obtained by others.

## Conclusions

Our results constitute formal evidence that insulin-producing β-cells, and other endocrine cells of the mature pancreatic islet, derive from ductal cells of the embryonic organ. Furthermore, the ability to trace the lineage of cells expressing *Muc1 *at different timepoints allows us to compare their differentiation potential before and after birth. We find that *Muc1*-expressing cells lose the capacity for islet differentiation postnatally, prior to the major increase in β-cell numbers that occurs in juvenile mice. These data add to an emerging model for control of β-cell mass, driven by developmentally-programmed neogenesis in the womb and physiologically-regulated proliferation after birth.

## Methods

### Targeting CreERT2 to the *Muc1 *locus

We followed the procedure of Wu et al. [41] to generate a *Muc1*^*IRES*-*CreERT*2-*neo *^(*Muc1*^*IC*2*neo*^) targeting vector. In brief, we recombineered a 9.2 kb fragment of the mouse *Muc1 *gene from a 129Sv BAC library (clone bMQ-356N19, from the Sanger Institute) into a conventional plasmid, introduced an *IRES-CreERT2-FRT-neo*^*R*^*-FRT *cassette after the endogenous Muc1 stop codon, and flanked the homology arms with *thymidine kinase *(*tk*) cassettes (Figure 3D). The targeting vector was then electroporated into R1 ES cells [42], generously provided by Mario Capecchi, which were selected with G418 and FIAU [43]. 52/96 of the surviving clones exhibited homologous recombination upon Southern blotting with a probe located outside the 5' homology arm. Proper recombination was confirmed for 8/8 of these upon further Southern analyses, and one of these clones, DK5.25 (Figure 3E), was used to generate chimeras (University of Utah, Transgenic Core Facility).

### Animal experiments

After initial backcrossing to C57BL/6, F1 offspring of a DK5.25 chimera (Figure 3E) were bred to *Rosa26*^*FLPo *^deletor mice [44], obtained from the Jackson Laboratory (Bar Harbor, ME), to delete the FRT-flanked *neo*^*R *^cassette. *neo*^*R *^excision yielded the *Muc1*^*IRES*-*CreERT*2 ^(*Muc1*^*IC*2^) allele (data not shown). Multiplex PCR genotyping, producing bands of 357 bp (wildtype) and 464 bp (mutant), was performed using oligos: wt forward: 5'-AATGGCAGTAGCAGTCTCTC-3'; wt reverse: 5'-CACAGCTGGCATAACTAACA-3'; and mutant reverse: 5'-CCACAACTATCCAACTCACA-3'. *Muc1*^*IC*2 ^mice were maintained on a CD1 outbred background. Cre reporter mice *Rosa26*^*EYFP *^[25] and *Rosa26*^*LacZ *^[26] were obtained from the Jackson Laboratory.

Tamoxifen (Sigma T-5648) was dissolved in corn oil, and administered by oral gavage at doses of 5-10 mg to adult mice, or 2 mg by subcutaneous injection of neonates. For timed-pregnancy studies, noon on the day after vaginal plugging was considered embryonic day 0.5 (E0.5). All animal procedures were approved by the Institutional Animal Care and Use Committee.

### Immunostaining and analysis

Tissue fixation, processing and immunostaining were performed essentially as described [45]. Tissues were fixed with 4% paraformaldehyde (PFA) in PBS for 1-2 hrs at 4°C, embedded in OCT and cryosectioned at 7-8 μm thickness. Primary antibodies used in this study are listed in Table 3. Secondary antibodies were purchased from Jackson Immunoresearch. To calculate labeling efficiencies, we photographed 5-12 randomly selected 20× fields per stained specimen, across 4-8 sections separated by 100-150 μm. The total number of each cell type (DAPI for total cells per field, LacZ or GFP for *Muc1*^*IC*2^-labeled cells, insulin and glucagon for endocrine cells, amylase, cytokeratin-19 and DBA lectin for exocrine cells) was determined using the Analyze Particles function of ImageJ (NIH). Double-positive cells were detected by additive image overlay, in ImageJ, of the DAPI channel with lineage^+ ^and marker^+ ^staining. Accuracy of counts was confirmed by eye in Adobe Photoshop for random samples. Calculations and graphs were generated with Microsoft Excel and R http://www.r-project.org.

**Table 3:** Primary antibodies used in this study

Antigen	Species	Source	Catalog #	Dilution
amylase	sheep	BioGenesis	0480-0104	1:2500
amylase	rabbit	Sigma	A8273	1:1000
cytokeratin-19	rat	Developmental Studies Hybridoma Bank	TROMA-3	1:50
cytokeratin-19	rabbit	Ben Stanger (University of Pennsylvania)		1:1000
C-peptide	rabbit	Linco	4020-01	1:2500
C-peptide	goat	Linco	4023-01	1:5000
E-cadherin	rat	Zymed/Invitrogen	13-1900	1:2000
GFP	rabbit	Abcam	ab290	1:4000
GFP	goat	Rockland	600-101-215	1:2500
glucagon	rabbit	Zymed/Invitrogen	18-0064	1:250
glucagon	guinea pig	Linco	4031-01F	1:2500
LacZ	rabbit	Cappel/MP	55976	1:2000
Muc1	hamster	NeoMarkers	HM-1630-P1	1:500
Neurog3	mouse	Developmental Studies Hybridoma Bank	I25A1B3	1:75

### Acinar isolation and staining

Acini were isolated by sequential trypsin and collagenase P digestion of minced dorsal pancreas, as described [46], PFA-fixed for 15 min and adhered to microscope slides by cytospin (Thermo-Fisher). Cytospin slides were stained as per cryosections. For wholemount immunofluorescence of intact tissue, small pieces of the dorsal pancreas were excised and PFA-fixed as above, washed with PBS, permeabilized with 1% Triton X-100 in PBS and stained with primary and secondary antibodies (overnight incubations, followed by extensive PBS/0.1% Tween-20 washes).

## List of Abbreviations

Cpa1: carboxypeptidase A1; CK19: cytokeratin-19; DAPI: 4',6-diamidino-2-phenylindole; DBA: Dolichos biflorus agglutinin; EYFP: enhanced yellow fluorescent protein; FIAU: 1-(-2-deoxy-2-fluoro-1-β-D-arabinofuranosyl)-5-iodouracil; GFP: green fluorescent protein; IRES: internal ribosome entry sequence; PFA: paraformaldehyde; TM: tamoxifen.

## Authors' contributions

LCM and DK developed the study concept and design. LCM provided input on methodology and analysis, and supervised the study. DK performed experiments, acquired and analyzed data. DK and LCM interpreted the data and wrote the manuscript. Both authors have read and approved the final manuscript.

## References

[B1] Bensley RR Studies on the pancreas of the guinea pig Am J Anat 1911 12 297 388 10.1002/aja.1000120304

[B2] Murtaugh LC Kopinke D The Stem Cell Research Community, StemBook Pancreatic stem cells (July 11, 2008) . Stembook 2008 http://www.stembook.org

[B3] Bonner-Weir S Weir GC New sources of pancreatic beta-cells Nat Biotechnol 2005 23 857 61 10.1038/nbt1115 16003374

[B4] Pictet R Rutter WJ Steiner D, Freinkel N Development of the embryonic endocrine pancreas Handbook of Physiology, Section 7 1972 1 Baltimore: Williams & Williams 25 66

[B5] Teta M Rankin MM Long SY Stein GM Kushner JA Growth and regeneration of adult beta cells does not involve specialized progenitors Dev Cell 2007 12 817 26 10.1016/j.devcel.2007.04.011 17488631

[B6] Finegood DT Scaglia L Bonner-Weir S Dynamics of beta-cell mass in the growing rat pancreas. Estimation with a simple mathematical model Diabetes 1995 44 249 56 10.2337/diabetes.44.3.249 7883109

[B7] Zhou Q Law AC Rajagopal J Anderson WJ Gray PA Melton DA A multipotent progenitor domain guides pancreatic organogenesis Dev Cell 2007 13 103 14 10.1016/j.devcel.2007.06.001 17609113

[B8] Desai BM J Oliver-Krasinski De Leon DD Farzad C Hong N Leach SD Stoffers DA Preexisting pancreatic acinar cells contribute to acinar cell, but not islet beta cell, regeneration J Clin Invest 2007 117 971 7 10.1172/JCI29988 17404620 PMC1838936

[B9] Gu G Dubauskaite J Melton DA Direct evidence for the pancreatic lineage: NGN3+ cells are islet progenitors and are distinct from duct progenitors Development 2002 129 2447 57 11973276 10.1242/dev.129.10.2447

[B10] Gradwohl G Dierich A LeMeur M Guillemot F neurogenin3 is required for the development of the four endocrine cell lineages of the pancreas Proc Natl Acad Sci USA 2000 97 1607 11 10.1073/pnas.97.4.1607 10677506 PMC26482

[B11] Schwitzgebel VM Scheel DW Conners JR Kalamaras J Lee JE Anderson DJ Sussel L Johnson JD German MS Expression of neurogenin3 reveals an islet cell precursor population in the pancreas Development 2000 127 3533 42 10903178 10.1242/dev.127.16.3533

[B12] Lee CS De Leon DD Kaestner KH Stoffers DA Regeneration of pancreatic islets after partial pancreatectomy in mice does not involve the reactivation of neurogenin-3 Diabetes 2006 55 269 72 16361411

[B13] Dor Y Brown J Martinez OI Melton DA Adult pancreatic beta-cells are formed by self-duplication rather than stem-cell differentiation Nature 2004 429 41 6 10.1038/nature02520 15129273

[B14] Brennand K Huangfu D Melton D All beta Cells Contribute Equally to Islet Growth and Maintenance PLoS Biol 2007 5 e163 10.1371/journal.pbio.0050163 17535113 PMC1877817

[B15] Bouwens L Rooman I Regulation of pancreatic beta-cell mass Physiol Rev 2005 85 1255 70 10.1152/physrev.00025.2004 16183912

[B16] Xu X D'Hoker J Stange G Bonne S De Leu N Xiao X Casteele M Van de Mellitzer G Ling Z Pipeleers D Beta cells can be generated from endogenous progenitors in injured adult mouse pancreas Cell 2008 132 197 207 10.1016/j.cell.2007.12.015 18243096

[B17] Ackermann Misfeldt A Costa RH Gannon M Beta-cell proliferation, but not neogenesis, following 60% partial pancreatectomy is impaired in the absence of FoxM1 Diabetes 2008 57 3069 77 10.2337/db08-0878 18728229 PMC2570403

[B18] Inada A Nienaber C Katsuta H Fujitani Y Levine J Morita R Sharma A Bonner-Weir S Carbonic anhydrase II-positive pancreatic cells are progenitors for both endocrine and exocrine pancreas after birth Proc Natl Acad Sci USA 2008 105 19915 9 10.1073/pnas.0805803105 19052237 PMC2604974

[B19] Means AL Xu Y Zhao A Ray KC Gu G A CK19(CreERT) knockin mouse line allows for conditional DNA recombination in epithelial cells in multiple endodermal organs Genesis 2008 46 318 23 10.1002/dvg.20397 18543299 PMC3735352

[B20] Solar M Cardalda C Houbracken I Martin M Maestro MA De Medts N Xu X Grau V Heimberg H Bouwens L Pancreatic exocrine duct cells give rise to insulin-producing beta cells during embryogenesis but not after birth Dev Cell 2009 17 849 60 10.1016/j.devcel.2009.11.003 20059954

[B21] Kushner JA Weir GC Bonner-Weir S Ductal origin hypothesis of pancreatic regeneration under attack Cell Metab 11 2 3 10.1016/j.cmet.2009.12.005 20085728 PMC3762572

[B22] Pierreux CE Poll AV Kemp CR Clotman F Maestro MA Cordi S Ferrer J Leyns L Rousseau GG Lemaigre FP The transcription factor hepatocyte nuclear factor-6 controls the development of pancreatic ducts in the mouse Gastroenterology 2006 130 532 41 10.1053/j.gastro.2005.12.005 16472605

[B23] Cano DA Murcia NS Pazour GJ Hebrok M Orpk mouse model of polycystic kidney disease reveals essential role of primary cilia in pancreatic tissue organization Development 2004 131 3457 67 10.1242/dev.01189 15226261

[B24] Feil R Wagner J Metzger D Chambon P Regulation of Cre recombinase activity by mutated estrogen receptor ligand-binding domains Biochem Biophys Res Commun 1997 237 752 7 10.1006/bbrc.1997.7124 9299439

[B25] Srinivas S Watanabe T Lin CS William CM Tanabe Y Jessell TM Costantini F Cre reporter strains produced by targeted insertion of EYFP and ECFP into the ROSA26 locus BMC Developmental Biology 2001 1 4 10.1186/1471-213X-1-4 11299042 PMC31338

[B26] Soriano P Generalized lacZ expression with the ROSA26 Cre reporter strain Nat Genet 1999 21 70 1 10.1038/5007 9916792

[B27] Ashizawa N Sakai T Yoneyama T Naora H Kinoshita Y Three-dimensional structure of peripheral exocrine gland in rat pancreas: reconstruction using transmission electron microscopic examination of serial sections Pancreas 2005 31 401 4 10.1097/01.mpa.0000181488.27399.dd 16258377

[B28] Schroeder JA Thompson MC Gardner MM Gendler SJ Transgenic MUC1 interacts with epidermal growth factor receptor and correlates with mitogen-activated protein kinase activation in the mouse mammary gland J Biol Chem 2001 276 13057 64 10.1074/jbc.M011248200 11278868

[B29] Balague C Audie JP Porchet N Real FX In situ hybridization shows distinct patterns of mucin gene expression in normal, benign, and malignant pancreas tissues Gastroenterology 1995 109 953 64 10.1016/0016-5085(95)90406-9 7657125

[B30] Herrera PL Huarte J Sanvito F Meda P Orci L Vassalli JD Embryogenesis of the murine endocrine pancreas; early expression of pancreatic polypeptide gene Development 1991 113 1257 65 1811941 10.1242/dev.113.4.1257

[B31] Means AL Meszoely IM Suzuki K Miyamoto Y Rustgi AK Coffey RJ Jr Wright CV Stoffers DA Leach SD Pancreatic epithelial plasticity mediated by acinar cell transdifferentiation and generation of nestin-positive intermediates Development 2005 132 3767 76 10.1242/dev.01925 16020518

[B32] Georgia S Bhushan A Beta cell replication is the primary mechanism for maintaining postnatal beta cell mass J Clin Invest 2004 114 963 8 15467835 10.1172/JCI22098 PMC518666

[B33] Jiang Y Nishimura W Devor-Henneman D Kusewitt D Wang H Holloway MP Dohi T Sabo E Robinson ML Altieri DC Postnatal expansion of the pancreatic beta-cell mass is dependent on survivin Diabetes 2008 57 2718 27 10.2337/db08-0170 18599523 PMC2551682

[B34] Wu X Wang L Schroer S Choi D Chen P Okada H Woo M Perinatal survivin is essential for the establishment of pancreatic beta cell mass in mice Diabetologia 2009 52 2130 41 10.1007/s00125-009-1469-6 19644667

[B35] Grupe A Hultgren B Ryan A Ma YH Bauer M Stewart TA Transgenic knockouts reveal a critical requirement for pancreatic beta cell glucokinase in maintaining glucose homeostasis Cell 1995 83 69 78 10.1016/0092-8674(95)90235-X 7553875

[B36] Kitamura T Kido Y Nef S Merenmies J Parada LF Accili D Preserved pancreatic beta-cell development and function in mice lacking the insulin receptor-related receptor Mol Cell Biol 2001 21 5624 30 10.1128/MCB.21.16.5624-5630.2001 11463843 PMC87283

[B37] Kushner JA Ciemerych MA Sicinska E Wartschow LM Teta M Long SY Sicinski P White MF Cyclins D2 and D1 are essential for postnatal pancreatic beta-cell growth Mol Cell Biol 2005 25 3752 62 10.1128/MCB.25.9.3752-3762.2005 15831479 PMC1084308

[B38] Rane SG Dubus P Mettus RV Galbreath EJ Boden G Reddy EP Barbacid M Loss of Cdk4 expression causes insulin-deficient diabetes and Cdk4 activation results in beta-islet cell hyperplasia Nat Genet 1999 22 44 52 10.1038/8751 10319860

[B39] Zhang H Ackermann AM Gusarova GA Lowe D Feng X Kopsombut UG Costa RH Gannon M The FoxM1 transcription factor is required to maintain pancreatic beta-cell mass Mol Endocrinol 2006 20 1853 66 10.1210/me.2006-0056 16556734

[B40] Nir T Melton DA Dor Y Recovery from diabetes in mice by beta cell regeneration J Clin Invest 2007 117 2553 61 10.1172/JCI32959 17786244 PMC1957545

[B41] Wu S Ying G Wu Q Capecchi MR A protocol for constructing gene targeting vectors: generating knockout mice for the cadherin family and beyond Nat Protoc 2008 3 1056 76 10.1038/nprot.2008.70 18546598

[B42] Nagy A Rossant J Nagy R W Abramow-Newerly Roder JC Derivation of completely cell culture-derived mice from early-passage embryonic stem cells Proc Natl Acad Sci USA 1993 90 8424 8 10.1073/pnas.90.18.8424 8378314 PMC47369

[B43] Nagy A Gertsenstein M Vintersten K Behringer R Manipulating the mouse embryo : a laboratory manual 2003 3 Cold Spring Harbor, N.Y.: Cold Spring Harbor Laboratory Press

[B44] Farley FW Soriano P Steffen LS Dymecki SM Widespread recombinase expression using FLPeR (flipper) mice Genesis 2000 28 106 10 10.1002/1526-968X(200011/12)28:3/4<106::AID-GENE30>3.0.CO;2-T 11105051

[B45] Murtaugh LC Law AC Dor Y Melton DA Beta-Catenin is essential for pancreatic acinar but not islet development Development 2005 132 4663 74 10.1242/dev.02063 16192304

[B46] Kurup S Bhonde RR Analysis and optimization of nutritional set-up for murine pancreatic acinar cells JOP 2002 3 8 15 11884762

